# Characterization of herpes simplex virus type 1 L-particle assembly and egress in hippocampal neurones by electron cryo-tomography

**DOI:** 10.1111/cmi.12093

**Published:** 2013-01-11

**Authors:** Iosune Ibiricu, Ulrike E Maurer, Kay Grünewald

**Affiliations:** 1 Department of Molecular Structural Biology, Max Planck Institute of BiochemistryMartinsried, D-82152, Germany; 2 Oxford Particle Imaging Centre, Division of Structural Biology, Wellcome Trust Centre for Human Genetics, University of OxfordOxford, OX3 7BN, UK

## Abstract

Visualizing virus–host interactions *in situ* inside infected cells by electron cryo-tomography provides unperturbed snapshots of the infection process. Here we focus on the assembly and egress pathway of herpesviruses. Cells infected with herpes simplex virus 1 produce and release not only infective virions but also non-infectious light particles (L-particles). L-particles are devoid of viral capsids and genomes. In this study, we analysed L-particle assembly and egress pathways in cultured dissociated hippocampus neurones by electron cryo-tomography. Virion and L-particle formation occurred in close proximity, suggesting shared assembly and exit pathways. Clathrin-like coats were occasionally associated with L-particle and virion assembly sites. Further, we compared the three-dimensional ultrastructure of intracellular and extracellular L-particles and quantified their diameters and the abundance of inclusion bodies contained.

## Introduction

Cells infected with herpes simplex virus 1 (HSV1) do not only produce infectious particles (virions) consisting of a genome-containing icosahedral capsid, which is surrounded by an amorphous layer of tegument proteins and a glycoprotein-rich lipid bilayer (Grünewald *et al*., 2003), but also non-infectious light particles (L-particles) characterized by the absence of capsids and viral DNA. When purifying viral preparations by density gradient centrifugation, virions and L-particles appear as a sharp lower band and a diffuse upper band, respectively (Szilagyi and Cunningham, 1991), hence their name. L-particles are present in all alphaherpesviruses tested so far (McLauchlan and Rixon, 1992; Dargan and Subak-Sharpe, 1997). They cover a wide range of sizes and often contain inclusion vesicles of variable size and number. Inclusion vesicles have been associated with certain phosphoproteins (Szilagyi and Berriman, 1994). Interestingly, L-particles were shown to facilitate the infection of HSV1 in cell culture, most likely by delivering additional tegument proteins to the target cell cytosol that are needed during viral replication (McLauchlan *et al*., 1992; Dargan and Subak-Sharpe, 1997) and by signalling events (reviewed in Meckes and Raab-Traub, 2011). It has been suggested that L-particles play also a role *in vivo*, e.g. in the formation of senile plaques in Alzheimer's diseased patients (Kammerman *et al*., 2006). L-particles are also present in significant amounts *in vivo* in swine nasal mucosa after pseudorabies virus infection (Aleman *et al*., 2003).

Little is known about the assembly mechanisms and exit pathway of L-particles. During virion assembly (secondary envelopment) progeny HSV1 capsids get enveloped in the cytosol by membranous compartments. These membranes originate from the trans-Golgi network and are associated with viral glycoproteins that can interact with a subset of tegument proteins (Loomis *et al*., 2001; Diefenbach *et al*., 2008; Nagel *et al*., 2008; Mettenleiter *et al*., 2009). Once enveloped, virions are transported to the plasma membrane and exit the cell by fusion of the membranous transport compartment with the plasma membrane (Mettenleiter *et al*., 2009). Previous electron microscopy studies of plastic embedded sections showed that L-particles might be assembled by budding of condensed tegument into Golgi-derived vesicles (Aleman *et al*., 2003). L-particle and virion assembly was previously observed simultaneously within the same enveloping compartment, suggesting that L-particles and virions share common assembly sites (Aleman *et al*., 2003). However, another study proposed that L-particles can be formed independently of virions, since cells infected at non-permissive temperatures with a temperature-sensitive HSV1 mutant produced L-particles but no virions (Rixon *et al*., 1992). L-particle characterization by electron microscopy studies of plastic embedded sections is limited by the fact that the specimen underwent chemical fixation, dehydration and heavy metal staining.

Using cellular electron cryo-tomography (cryoET) (Lučić *et al*., 2005; Yahav *et al*., 2011) we here compared the assembly and egress pathways of HSV1 L-particles and virions. This technique allowed us to visualize, at a macromolecular level and in three dimensions (3D), L-particle assembly and exit *in situ* under close-to-native conditions.

## Results and discussion

**Fig. 1:**
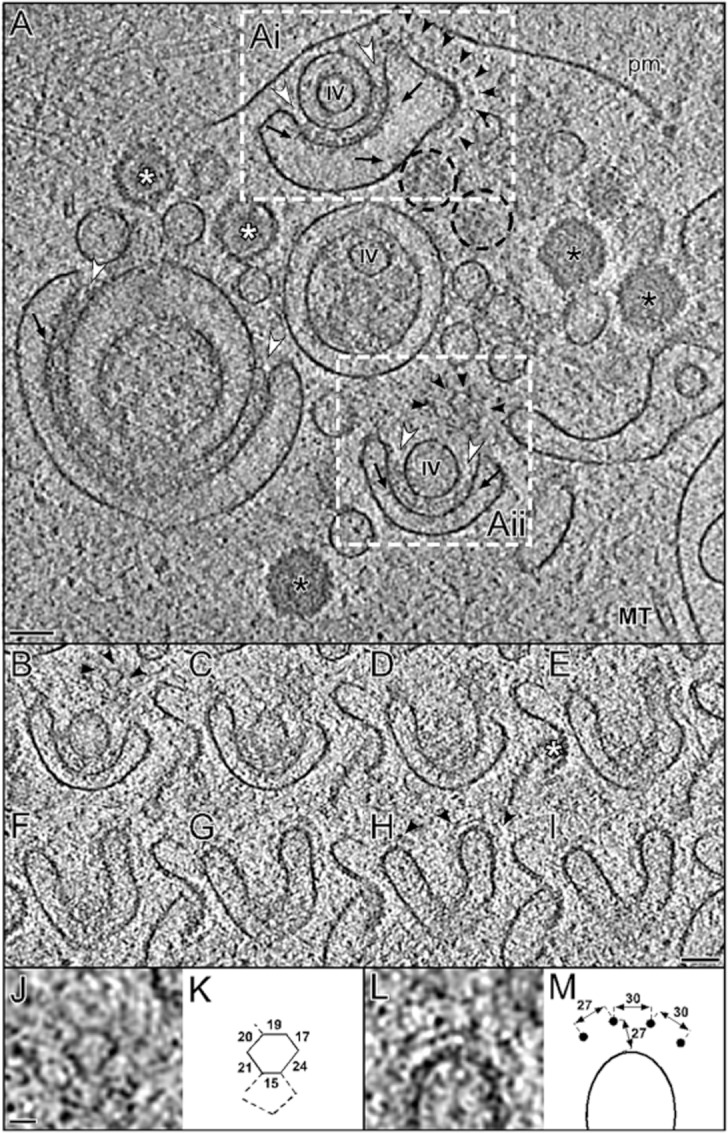
Electron cryo-tomographic reconstruction of an HSV1 assembly site in an axon terminal of a vitrified hippocampal neurone. A. Computational slice showing L-particle assembly events marked by rectangles Ai and Aii. Inclusion vesicles (IV) are enveloped by membranous compartments associated with characteristic densities, presumably glycoproteins (black arrows) on their lumenal face and tegument proteins (white arrowheads) on their cytosolic face. C-capsids (black asterisk) and B-capsids (white asterisk or black dashed circles for top views). MT: microtubule; pm: plasma membrane. Notably, a regular coat layer is clearly visible on the membranous compartment (black arrowheads). Scale bar: 100 nm. B–I. Parallel slices of 9.72 nm thickness through the area marked in Aii. Coat scaffolds are visible at the edge of the compartment (black arrowheads). The hexagonal-pentagonal arrangement of the scaffold coat is clearly recognized in (B) as well as the side view (H). Scale bars: 80 nm. For the tomographic volume of (B–I), see Supporting Movie S1. J, K and L, M. Close-ups and schematic drawings of (B) and (H) respectively. The numbers in (K) and (M) represent distances in nm. Scale bars: 20 nm.

Dissociated hippocampal neurones growing on holey carbon support films on electron microscopy grids were infected with HSV1 strain F and vitrified at 16 h p.i. as described (Ibiricu *et al*., 2011). At this time point we observed L-particle assembly sites in axon terminals of hippocampal neurones (Fig. 1). These sites were characterized by the presence of capsids and membranous compartments associated with tegument- and glycoprotein-like densities. Not only capsids with DNA (C-capsids) were found, but also capsids devoid of DNA (A- and B-capsids) (Newcomb and Brown, 2008; Heymann *et al*., 2003; Ibiricu *et al*., 2011). Furthermore, vesicles (future inclusion vesicles) were likewise found undergoing envelopment. The origin of these vesicles is not known, although an origin identical to that of the enveloping compartment seems likely. Consistent with this, the inclusion vesicles showed characteristic lumenal densities. Some of these densities connected to the membrane, closely resembled glycoprotein spikes observed during virion envelopment (Ibiricu *et al*., 2011). Tomographic snapshots of the envelopment process showed for the membranous compartments a characteristic concave curvature at the sites of interaction with inclusion vesicles (Fig. 1, Supporting Movie S1). We observed characteristic accumulation of densities in between the inclusion vesicles and the enveloping compartments. The appearance and fine structure of these densities was highly similar to those of tegument densities described in cryoET studies of virion secondary envelopment (Ibiricu *et al*., 2011). Therefore, we attributed the densities between the inclusion vesicles and the enveloping compartment to tegument proteins. Moreover, spike-like densities, presumably glycoproteins, were associated to the lumenal side of the enveloping compartment with the highest density being at the sites of active envelopment. Double envelopment of L-particles including their enveloping membrane (Fig. 1A left) and, occasionally, inclusion vesicles containing themselves vesicle like inclusions was also observed (Fig. 1Ai). In the latter case, the formation of the inner vesicle is topologically identical to that of L-particle formation without inclusion body, i.e. the content of the inner vesicle constitutes cytosol. In the case of Fig. 1Ai the inner vesicle seems relatively small, possibly the result of an aborted L-particle formation. Double envelopment can be explained by interactions of tegument proteins that were themselves both linked to two different membranes via a cascade of interaction: (i) between tegument proteins and (ii) tegument proteins with the glycoprotein cytoplasmic tails. Why double envelopment was not observed for already enveloped capsids remains to be revealed in detail. One explanation might be that inclusion vesicle envelopment is less controlled or slower than that of capsids and therefore prone to a wider range of outcomes.

Intriguingly, some of the surfaces of the membranous compartments enveloping the L-particles and capsids, typically the most convex surfaces, had on the cytosolic side a distinctive coat (Fig. 1, Supporting Movie S1). This coat extended max ∼ 25 nm from the membrane and was regularly arranged forming pentagons and hexagons of ∼ 30 nm maximum width. This arrangement resembles clathrin cages (Kirchhausen, 2000; Fotin *et al*., 2006; Cheng *et al*., 2007). Clathrin-coated vesicles are typically formed in the trans-Golgi network, on endosomes and at the plasma membrane (Kirchhausen, 2000; Spang, 2008). Clathrin coats are assembled from clathrin triskelia and form a characteristic pentagonal and hexagonal lattice (Kirchhausen, 2000; Cheng *et al*., 2007; Spang, 2008). Although COP I coats were earlier suggested to form a pentagonal-hexagonal arrangements (Hughson, 2010; Lee and Goldberg, 2010), a recent cryoET study of COPI-Coated vesicles revealed a 14-nm-thick coat of triads (Faini *et al*., 2012). Therefore the here observed coat on the membranous enveloping compartments could be formed by clathrin. This is further consistent with the fact that the HSV1 enveloping compartment originates at the trans-Golgi network while COP I coats are mostly present on vesicles transported between the endoplasmic reticulum and early secretory compartments (Bethune *et al*., 2006). Although we found coat scaffolds occasionally also in less curved membrane areas, they predominantly localized to the areas of the enveloping compartment where the membrane had the highest curvature (Fig. 1Ai, Aii). Clathrin and other scaffold coats are known to enhance the membrane curvature (Alberts *et al*., 2007). Our findings suggest a possible function of coat scaffolds, most likely clathrin, in secondary envelopment. To elucidate a potential significant interaction between HSV1 assembly and clathrin further, we performed live cell spinning disk confocal imaging of clathrin-labelled BSC-1 cells infected with HSV1-GFPVP26. However, colocalization between the tagged HSV1 small capsid protein and clathrin was infrequent. Therefore, it remains ambiguous whether clathrin is significantly involved in HSV1 assembly and cell type dependence should be considered in follow-up studies.

**Fig. 2:**
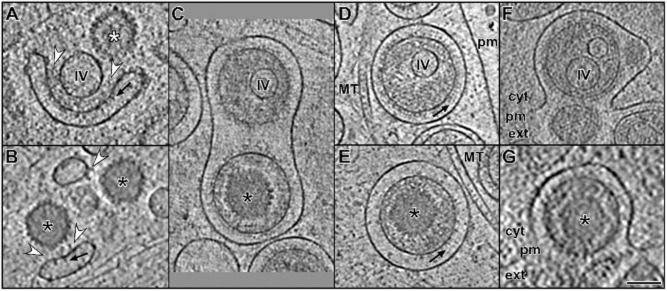
Structural comparison between L-particle and virion assembly and egress in cryo-ET. Slices through tomographic volumes showing secondary envelopment and exit of HSV1 L-particles (upper row) and virions (lower row). A and B. At axon terminals, membranous compartments associated with glycoproteins (black arrows) and tegument (white arrowheads) surround inclusion vesicles (IV) and capsids (black and white asterisks for C- and B-capsids respectively). (B) is a zoom into fig. 6C from Ibiricu *et al*. (2011). C. L-particle and virion sharing a membranous compartment in an axon terminal. Black arrows: glycoproteins; MT: microtubule; pm: plasma membrane; black asterisk: C-capsid. D and E. L-particle and virion from the same tomogram in a middle region of an axon. F and G. Exit by fusion of the membranous compartment with the plasma membrane (pm) at an axon terminal. Black asterisk: C-capsid; cyt: cytoplasm; ext: extracellular space; MT: microtubule; pm: plasma membrane. Scale bar for (A)–(G): 100 nm.

Overall, L-particles and virions apparently follow an identical egress pathway. Assembly in axon terminals occurred by envelopment of capsids or inclusion vesicles with a membranous compartment (Fig. 2A and B). Capsid or inclusion vesicle interaction with tegument proteins and between tegument and glycoproteins seemed to play an important role in mediating this process (Fig. 2A and B). In strong support for an identical assembly and egress pathway is our observation that assembled L-particles and virions in some cases even shared the same membranous compartment (Fig. 2C). They also appeared inside independent compartments next to each other and close to microtubules in middle regions of axons (Fig. 2D and E). Finally, both L-particles and virions exited the cell at axon terminals by fusion of the membranous compartment with the plasma membrane (Fig. 2F and G).

The 12 acquired tomograms from hippocampal neurones contained a total of 47 fully enveloped intracellular L-particles. They were recognized as enveloped vesicles containing electron dense material, resembling tegument protein densities in virions, and in some cases inclusion vesicles. The vesicle surface was studded with spikes resembling glycoprotein spikes on virions.

**Fig. 3:**
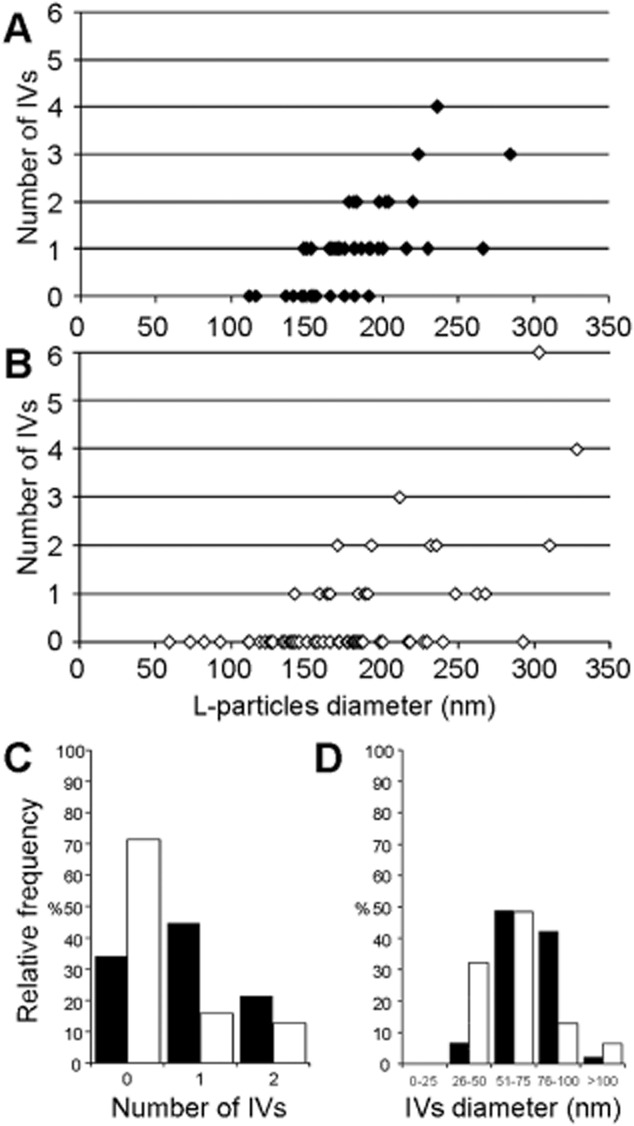
Comparison between intracellular L-particles from neurones (black) and purified L-particles from a viral preparation (white). A and B. L-particle distribution depending on the L-particle diameter and the number of inclusion vesicles (IVs) in the particle. C. L-particle distribution depending on the number of inclusion vesicles per particle. D. Inclusion vesicle distribution depending on the inclusion vesicle diameter.

We next compared the morphology of intracellular L-particles to extracellular ones found in virus preparations (Döhner *et al*., 2006) (Fig. 3). Since isolation of viral particles from neurones is highly inefficient, viral particles were produced in human foreskin fibroblasts. Sixty-two L-particles were selected from 24 tomograms of vitrified viral preparations. Intracellular L-particles were nearly spherical while isolated L-particles were more heterogeneous in size and shape, probably due to purification. Thus, only extracellular L-particles with a relatively spherical shape were considered. The average diameter of intracellular and extracellular L-particles was 180 nm and 177 nm respectively. This is bigger than previously reported for isolated L-particles explored by studying 2D projections (Szilagyi and Berriman, 1994). Two-thirds of the intracellular L-particles, but only 30% of the extracellular ones, contained one inclusion vesicles or more (Fig. 3C). The reason for this might be in the different cell types used. Inclusion vesicles were typically eccentrically located within the particle (Fig. 2D), as previously described for virion capsids (Grünewald *et al*., 2003; Maurer *et al*., 2008). Especially for smaller inclusion vesicles their lumen density differed from tegument in its texture, consistent with previous studies describing the inclusion vesicle components as polypeptides unique to L-particles (Szilagyi and Cunningham, 1991; Szilagyi and Berriman, 1994). In disagreement to earlier studies (Szilagyi and Berriman, 1994) we occasionally observed both inclusion vesicles and capsids in the same virion (data not shown) indicating that inclusion vesicles are not exclusive to L-particles.

In conclusion, cryoET allowed us to characterize L-particle morphology, assembly and egress. Inside neurones, virion and L-particle formation occurred in close proximity and even enveloped in the same membranous compartment, suggesting a shared assembly and exit pathway. Our novel observation of a coat scaffold during viral assembly and egress in neurones demands further analysis of its role. By analysing the structure of L-particles in 3D we were able to exactly determine the number of inclusion vesicles present. The specific role of inclusion vesicles is unclear; putatively they might facilitate L-particle formation and envelopment in neurones infected with HSV1 and the fact that L-particles share the assembly and egress pathway with virions, ensures their formation alongside virions – a prerequisite to fulfil their role during infection (Dargan and Subak-Sharpe, 1997).

## Experimental procedures

### Viral preparation

HSV1 virions (wild-type strain F) were amplified in BHK-21 cells or human foreskin fibroblasts and the viral titre was determined by plaque titration on Vero cells as described previously (Döhner *et al*., 2006). Virus stocks used for infection of cultured neurones used had a titre of 10^9^ PFU ml^−1^.

### L-particle preparation

Characterization of L-particle morphology and statistical analysis of the number of inclusion vesicles were performed from L-particles co-purified with gradient purified virions. In brief, medium supernatant from infected human foreskin fibroblasts was harvested 70 h p.i., low speed clarified from cell debris and virus particles were pelleted. After swelling in buffer overnight at 4°C the pellet was gently resuspended. This raw preparation was further purified by Nycodenz gradient centrifugation and the virion band harvested (Döhner *et al*., 2006).

### Infection of neurones on grids

Cell culture and infection of neurones on EM grids was essentially done as reported previously (Ibiricu *et al*., 2011). In brief, Au grids of 200 mesh with holey carbon support films (2/1, Quantifoil GmbH, Jena, Germany) were sterilized under UV light and then coated with poly-l-lysine (Sigma) overnight. Grids were then washed and placed into neurobasal medium (Gibco) supplemented with B27 (Gibco) and glutamine. Hippocampal neurones (kindly provided by F. Bradke, MPI Neurobiology, Germany) were isolated from 17-day-old rat embryos. Dissociated neurones were plated over the grids at a density of 100 000 cells in a 60-mm-diameter Petri dish and incubated 7 days at 37°C and 5% CO_2_ to enable the growth of axons and dendrites. They were then infected with HSV1 at an moi of 50 PFU per cell. Infected cells were further incubated at 37°C, 5% CO_2_ for 16 h.

### Preparation of specimens for cryo-EM

For cellular specimens grown on grids, grids were removed from the cell culture dish and 2 μl of a colloidal gold suspension (coated with BSA, 10 nm diameter, in HBSS buffer) were added immediately before removing excess of liquid by blotting the grid with a filter paper. Specimens were then vitrified by plunge-freezing into liquid ethane and transferred into liquid nitrogen for storage. For purified L-particles 2 μl of the participle preparation were applied to glow discharged holey carbon support films (2/1, Quantifoil GmbH, Jena, Germany) on copper grids. Then, 2 μl of the colloidal gold markers in buffer were added and samples blotted and vitrified as for the cellular specimens.

### Data collection by cryo-ET

Data were collected on a Tecnai F30 ‘Polara’ (FEI, Eindhoven, The Netherlands) transmission electron microscope equipped with a GIF 2002 postcolumn energy filter (Gatan, Pleasanton, CA). The microscope was operated at 300 keV and the energy filter in zero-loss mode with a slit width of 20 eV. Images (2048 × 2048 pxls) were collected on the CCD camera of the GIF 2002. For the chosen magnification the calibrated pixel size at the specimen level was 0.81 nm. Tilt series were collected from −60° to 60° with an angular increment of 2° and an exposure time scheme of 1/cos(α) of the tilt angle (α). Defocus was measured along the tilt axis after each tilt and automatically maintained at −12 (± 0.5) μm. The total electron dose received at the specimen level was kept between 60 and 90 electrons A^−2^.

### Image processing

Tomographic series of tilted images were aligned using 10 nm gold beads as fiducial markers. Three-dimensional reconstructions were calculated in IMOD (Kremer *et al*., 1996), TOM-toolbox (Nickell *et al*., 2005) and EM (Hegerl, 1996). Measurements of coat lattices and visualization (Gaussian filtering of volumes, snapshots of slices and movies through volume slices) were performed using the AMIRA software package (VSG, Merignac, France).

## Supporting information

**Movie S1.** Movie of the tomographic volume for the area shown in Fig. 1Aii and Fig. 1B–I detailing the membrane continuity and the 3D relative position of the inclusion vesicle and a capsid to the enveloping membrane compartment.
